# Comprehensive Analyses of Nitric Oxide-Induced Plant Stem Cell-Related Genes in *Arabidopsis thaliana*

**DOI:** 10.3390/genes10030190

**Published:** 2019-03-01

**Authors:** Muhammad Shahid, Qari Muhammad Imran, Adil Hussain, Murtaza Khan, Sang Uk Lee, Bong Gyu Mun, Byung-Wook Yun

**Affiliations:** 1 Laboratory of Plant Functional Genomics, School of Applied Biosciences, Kyungpook National University, Daegu 41566, Korea; shahidariswat@gmail.com (M.S.); mimranbot@gmail.com (Q.M.I.); murtazakhan.bio@gmail.com (M.K.); uk0gam@gmail.com (S.U.L.); 2 Department of Agriculture Abdul Wali Khan University, Mardan 23200, Pakistan; adilhussain@awkum.edu.pk

**Keywords:** *Arabidopsis thaliana*, stem cells, basal defense, promoter analysis, gene ontology

## Abstract

Plant stem cells are pluripotent cells that have diverse applications in regenerative biology and medicine. However, their roles in plant growth and disease resistance are often overlooked. Using high-throughput RNA-seq data, we identified approximately 20 stem cell-related differentially expressed genes (DEGs) that were responsive to the nitric oxide (NO) donor S-nitrosocysteine (CySNO) after six hours of infiltration. Among these DEGs, the highest number of positive correlations (*R* ≥ 0.8) was observed for *CLAVATA3*/*EMBRYO SURROUNDING REGION-RELATED* (*CLE*) 12. Gene ontology (GO) terms for molecular function showed DEGs associated with signal transduction and receptor activity. A promoter study of these DEGs showed the presence of *cis*-acting elements that are involved in growth as well as the regulation of abiotic and biotic stress. Phylogenetic analysis of the *Arabidopsis* stem cell-related genes and their common orthologs in rice, soybean, poplar, and tomato suggested that most soybean stem cell-related genes were grouped with the *Arabidopsis* CLE type of stem cell genes, while the rice stem cell-related genes were grouped with the *Arabidopsis* receptor-like proteins. The functional genomic-based characterization of the role of stem cell DEGs showed that under control conditions, the *clv1* mutant showed a similar phenotype to that of the wild-type (WT) plants; however, under CySNO-mediated nitrosative stress, *clv1* showed increased shoot and root length compared to WT. Furthermore, the inoculation of *clv1* with virulent *Pst* DC3000 showed a resistant phenotype with fewer pathogens growing at early time points. The qRT-PCR validation and correlation with the RNA-seq data showed a Pearson correlation coefficient of >0.8, indicating the significantly high reliability of the RNA-seq analysis.

## 1. Introduction

Stem cells are pluripotent cells that have gained the attention of the scientific community in the recent past because of their tremendous applications in the biomedical field, specifically in cancer and organ regeneration. However, it was out of our notice that stem cells are an integral part of our life as they are the ultimate precursors of most of the life-saving commodities such as food, fuel, and the oxygen we breathe [1]. Therefore, plant stem cells are the most important cells affecting human life directly or indirectly. Despite concrete differences in cellular biophysics and the properties between animal and plant cells, the basic concept and mechanism of action of their stem cells is curiously the same [1]. Interestingly, the cellular and molecular design of these systems are conserved across the species. Another reason for plant stem cells being more important is that they might remain active for a long time, even over hundreds or thousands of years, suggesting a fine-tuned, well self-maintained underlying proliferation and differentiation mechanism. 

As the major producers of the ecosystems collecting and consuming solar energy, plants do not need to hunt for their food requirement; rather, they need to increase their surface area to capture more energy, and thus are sessile [1]. Consequently, they have to deal with several environmental insults, including herbivory and pathogen attack. Plants have evolved a mechanism to cope with these adversities; one such strategy is a post-embryonic mode of development that makes plants able to regenerate their organs during the course of their life. The most important components of this developmental strategy are pluripotent stem cells that are permanently active and embedded in specialized tissues called meristems. These specified tissues are located at growth points, such as shoot and root apices in plants, whose fates are decided by their positions and can be adapted according to environmental conditions [2]. 

The *Arabidopsis* shoot apical meristem (SAM), as the name indicates, is located at the shoot apex; it is a dome-shaped tissue made of small cells that are defined and maintained by the expression of the homeodomain transcription factor *SHOOTMERISTEM-LESS* (STM) [3,4]. *STM* is responsible for the suppression and maintenance of the proliferative capacity of meristematic cells [5]. The inductive niche of *STM*, which is called the organizing center (OC), is located basally to the stem cells, and is defined by the expression of the homeodomain transcription factor *WUSCHEL* (*WUS*), which is required for stem cell maintenance [6,7]. Other important genes that are responsible for regulating SAM stem cell fate are the *CLAVATA* (*CLV*) genes, which are involved in the regulation of OC and *STM*. The activation of the *CLV3*-dependent signaling pathway decreases stem cell proliferation and induces organ initiation through a feedback loop that inhibits *WUS* expression [8,9]. The gain-of-function studies involving 18 different *Arabidopsis CLE* genes demonstrated functional redundancy, often opposing their phenotypes such as increased root and rosette development, stunting, dwarfing, SAM arrest, perturbed leaf development, and “shrub-like” phenotypes in various combinations [10]. These pleiotropic phenotypes were attributed to integrated hypermorphic and global neomorphic responses to abundant ectopic ligands through multiple signaling pathways [10]. The functional redundancy of the CLE peptides was also shown in another study involving the exogenous application of 26 chemically synthetic CLE peptides, corresponding to the 31 *Arabidopsis CLE* gene products [11]. These synthetic peptides, when applied to *Arabidopsis* and rice, showed variable phenotypic effects on roots and SAM [11,12]. Relatively high sequence conservation is found between various *Arabidopsis* CLE proteins, and are expressed in various plant tissues [13]. The CLE peptides regulate the growth and development of root and shoot meristematic cells. The plant pathogenic nematode *Heterodera schachtii* utilizes this function of CLE peptides to its own advantage by secreting the CLE mimics, HsCLE1 and HsCLE2, into the host system, thereby reprogramming the host cellular machinery for the formation of multinucleated feeding cells. Both these mimics share a highly conserved C-terminal CLE domain with *Arabidopsis* CLEs 1–7. The overexpression of these nematode mimics resulted in typical *wuschel*-like phenotypes, which were abolished when the proteins were expressed without the CLE-motif [12]. Significantly high sequence similarity has been observed between the CLE proteins of *Arabidopsis* and rice, some of which contain multiple CLE domains [14]. The *Arabidopsis* CLE gene family contains at least 44 members. The detailed multi-database search conducted in the present study also showed the presence of this gene family in other plant species, such as pepper (*Capsicum annuum*), poplar (*Populus trichocarpa*), potato (*Solanum tuberosum*), rice (*Oryza sativa*), and soybean (*Glycine max*). However, only a limited number of genes have known functions and are mainly involved in the maintenance of shoot apical meristem (SAM).

Nitric oxide (NO) emerged as a new signaling molecule in the recent past, and attracted the attention of biological researchers because of its diverse roles in both animal and plant systems [15,16]. Since its discovery in plants, NO has been reported to regulate a plethora of biological processes, such as germination [17], flowering time [18], flower development [19], apical dominance [19,20], and auxin-mediated root growth and development [21]. Similarly, the effects of NO on internode elongation and hypocotyl emergence have also been studied. In a study using NO donors, it was observed that NO inhibited hypocotyl growth and enhanced de-etiolation and chlorophyll in *Arabidopsis* and potato [17]. Reports also suggested the role of NO in the induction of adventitious roots development in cucumber [22], whereas the NO scavenger, 2-4-carboxyphenyl-4,4,5,5-tetramethylimidazoline-1-oxyl-3-oxide (cPTIO), inhibited its formation. Previous studies also suggested the role of NO in maize root elongation [23]. In a recent study, we also discovered the role of the NO-responsive *RAPID ALKANIZATION FACTOR* (*RALF*) genes in root growth and development. These genes showed a negative role in growth and development as the *ralfl1* and *ralfl22* mutants produced longer roots compared to the wild type [24]. 

The role of NO in plant defense is well explored. Sensing avirulent microbial pathogen activates the production of reactive oxygen intermediates (ROIs) and reactive nitrogen species (RNS), which play important roles in the plant defense system. An avirulent pathogen infection can start a network of inducible defense mechanisms in both local and systemic plant tissues [25]. Concluding all this, NO plays diverse roles in plant growth, development, and defense responses; however, the exact mechanism of growth regulation is yet to be understood. Therefore, the increasing evidence of the production and deleterious effects of RNS along with ROIs is gaining increasing importance day by day, and need to be explored further. In a recent study, using the RNA-seq mediated transcriptomic analysis, we identified global changes in gene expression in response to one of the common NO donors, S-nitrosocysteine (CySNO), and found that NO regulates diverse biological processes [26]. In the current study using the high-throughput RNA-sequencing data, we identified approximately 20 stem cell and stem cell-related genes that showed differential expression to one mM of CySNO. By using both in silico and in vivo approaches, we suggested that the NO-responsive stem cell-related genes are mostly involved in plant growth; however, the promoter analysis suggests their putative roles in plant defense as well. This study might help scientists divert their attention toward creating strategies to use pluripotent stem cells in plant defense.

## 2. Materials and Methods

### 2.1. Transcriptome-Wide Identification and Characterization of Plant Stem Cell Genes

A number of DEGs that showed significant differential expression in response to one mM of CySNO were identified. A detailed list of those DEGs has been reported earlier [26]. Here, we manually searched for the presence of stem cell or stem cell-related genes in the list and found approximately 20 stem cell genes that showed significant (*p* ≤ 0.05) differential expression to CySNO. The list was carefully checked for any duplicate values. A heatmap with a dendrogram representing hierarchical clustering was generated to visualize the differences in expression values between the treated and control samples using the fragments per kilobase of transcript per million mapped reads (FPKM) values of the control and CySNO-treated samples using R version 3.3.1.R (https://www.r-project.org/). A principal component analysis (PCA) plot was also generated for the same samples to find any dispersion in the data using R.

### 2.2. Correlation and Gene Ontology Analyses

All of the NO-induced stem cell genes were then processed using the Phytomine tool of the Phytozome database (https://phytozome.jgi.doe.gov/pz/portal.html) in order to find the correlations among these genes. We identified both positive and negative correlations, which were visualized with red and blue colors, respectively. To further understand the regulatory role of the NO-induced plant stem cells, an analysis of the associated gene ontology (GO) terms for the biological processes, molecular functions, and cellular components was performed using the web interface of PANTHER version 11.1 (http://pantherdb.org), as described earlier [27]. Briefly, the locus IDs of the 20 differentially expressed NO-induced stem cell-related genes were searched using the appropriate search field, while *Arabidopsis thaliana* was selected as the organism of choice. The “statistical overrepresentation test” was selected as the method of analysis with default settings. The analyzed data for all the GO terms; that is, GO biological process, molecular function, and cellular location, were observed, and the GO terms with an enrichment *p*-value < 0.05 were downloaded for each annotation dataset and compiled using Microsoft Excel for quantitative presentation.

### 2.3. Promoter Analysis

Promoters play a key role in the regulation of genes, as they have specific sites for binding transcription factors that have the mechanistic control of gene transcription. Therefore, we sought to predict the regulatory roles of the NO-induced stem cell genes. For this, the promoter sequences of the CySNO-induced stem cell-related genes 1.5 kb upstream of their transcription initiation site were retrieved from The *Arabidopsis* Information Resource (TAIR) center (https://www.arabidopsis.org/). All of the sequences were analyzed for regulatory elements using the Plant Cis-acting Regulatory Elements (PlantCARE) web interface (http://bioinformatics.psb.ugent.be/webtools/plantcare/html/) [28]. The identified regulatory elements were manually screened, and only some of those involved in abiotic and biotic stresses were mapped using the Regulatory Sequence Analysis Tool [29].

### 2.4. Phylogenetic Analysis

To determine the evolutionary relationship among the stem cell-related genes from different species, the *Arabidopsis* stem cell protein sequences were queried against the protein sequences of rice (*Oryza sativa*), soybean (*Glycine max*), tomato (*Solanum lycopersicum* L.), and poplar (*Poplar trichocarpa*) in the Phytozome database, and only the proteins that were common in all the species were selected. We found approximately 28 orthologs in rice, 21 in soybean, four in tomato, and 15 in poplar. The protein sequences of these proteins were retrieved from Phytozome and aligned using the ClustalW tool; the resulting alignment was then used to generate a phylogenetic tree using the maximum likelihood method based on the Jones Taylor- Thornton (JTT) matrix-based model [30]. All of the positions having less than 95% site coverage were eliminated; that is, fewer than 5% alignment gaps, missing data, and ambiguous bases were allowed at any position. All of the evolutionary analyses were conducted using MEGA7 [31]. 

### 2.5. Plant Material and Growth Conditions

To further validate the roles of the NO-induced stem cell-related genes in plant growth and defense, we selected the *CLAVATA* genes for functional genomics study using the reverse genetics approach because of their defining roles in maintaining OC. Among the *CLAVATA* family genes, *CLV1* and *CLV3* are considered to be the most important ones, as they are involved in a feedback loop to maintain OC; therefore, we selected *CLV1* and *CLV3* for downstream analysis. The *Arabidopsis thaliana* seeds, including the wild-type seeds in Landsberg *erecta* (L*er*-0) and the Columbia 0 background (Col-0) and mutant lines *clv1* (CS37, At1G75820) and *clv3* (CS8066, At2G27250), were obtained from the Nottingham *Arabidopsis* Stock Centre (http://arabidopsis.info/BasicForm), and those with the *enhanced disease susceptibility 1-2* (*eds1-2*) gene were kindly provided by Prof. Gary J. Loake, Edinburgh University, United Kingdom. The *atgsnor1-3*(*At5g43940*) [32] and *cat2* (*At4g35090*) [33] were used for comparison as sensitive controls for nitrosative and oxidative stresses, respectively. The *CLAVATA* mutants and *eds1-2* were in the L*er* genetic background, while the remaining mutants were in the Col-0 background. All of the seeds were sterilized in 50% bleach solution for five minutes, incubated with 1% (*v*/*v*) TritonX-100, and rinsed with sterile distilled water three times. Then, the seeds were stratified at 4 °C overnight prior to sowing either on ½ Murashige and Skoog (MS) medium or in soil under long-day growth conditions (16 hours of light and eight hours of darkness at 23 ± 2 °C).

### 2.6. Redox Stress Assay

Plants were exposed to oxidative and nitrosative stresses, as described previously [24]. In brief, the plants were grown on ½ MS medium supplemented with 0.75 mM of S-nitrosoglutathione (GSNO) and 0.75 mM of S-nitrosocysteine (CysNO) for nitrosative stress and with two mM of H_2_O_2_ and one mM of methyl viologen for oxidative stress conditions. The control plants were grown only on ½ MS medium. The data on seed germination, cotyledon development frequency (CDF), and root and shoot lengths were recorded at appropriate intervals until 14 days of sowing (unless stated otherwise). The CDF was used for green developed seedlings, and was calculated as described earlier [34].

### 2.7. Pathogen Inoculation and Pathogenicity Assessment

To evaluate the response of *CLV* genes in basal defense, the wild-type and mutant lines were subjected to virulent *Pseudomonas syringe* pv. *tomato* (*Pst*) strain DC3000 inoculation. The pathogen was maintained and inoculated according to Yun et al. [35]. In short, the pathogen was grown on Luria–Bertani (LB) agar medium supplemented with 100 mg/mL of rifampicin as the antibiotic for selection and incubated at 30 °C overnight. Then, single colonies were then used to inoculate the LB broth that had rifampicin, and it was incubated at 30 °C with shaking overnight. The overnight-grown liquid culture was then centrifuged at 8000 rpm for three minutes, and the bacterial pellet was re-suspended in 10 mM of MgCl_2_ solution. The concentration of the cells was adjusted to 5 × 10^5^ colony-forming units (CFUs), and the cell suspension was infiltrated into the abaxial side of the leaves. The control inoculant contained 10 mM of MgCl_2_ only. The inoculated leaf samples were collected at zero hours to three days post-inoculation for bacterial colony counts and subsequent gene expression analysis. 

Plants were assessed phenotypically for any symptom development due to pathogen growth over time and photographed using a digital single-lens reflex (DSLR) Canon camera. For pathogenicity assessment, the leaf discs from the inoculated leaves, which were one cm in diameter, were homogenized in one mL of 10 mM of MgCl_2_ solution, and then subjected to serial dilution. The diluted homogenate was spread on the LB medium containing rifampicin and incubated under optimal growth conditions. The log colony-forming unit (CFU) was calculated using the dilution factor and the number of colonies grown.

### 2.8. q-RT PCR and Gene Expression

Leaf samples from the plants inoculated with *Pst* DC3000 were used to extract RNA using the Trizol^®^ (Invitrogen, USA) method according to the description by Imran et al. [36]. The RNA was used to synthesize the first strand of cDNA using the reverse transcriptase (RT) kit (BIOFACT, Korea) according to the manufacturer’s recommendations. The cDNA that was synthesized was used as a template for gene expression analysis. A 20-µL reaction mixture containing 2X Real-Time PCR Master Mix [(including SYBR^®^ Green I) BIOFACT, Korea] with 10 nM of each primer was processed in a two-step PCR reaction using the Eco™ real-time PCR (Illumina, USA). A “no-template control” containing nuclease-free water instead of the cDNA template was used as the negative control. The thermal cycling conditions included initial denaturation at 95 °C for 15 minutes, followed by 40 cycles of 95 °C for 10 seconds and 60 °C for 30 seconds. Actin was used as the internal reference gene. 

For validating the RNA-seq mediated transcriptional changes in stem cell-related genes, the wild type Col-0 plants were infiltrated with one mM of CySNO, and the samples were collected at zero and six hours post-infiltration. For validation, we selected a few stem cell-related genes that showed at least a fourfold change in gene expression when treated with one mM of CySNO after six hours of infiltration in RNA-seq analysis [26]. RNA extraction and qRT-PCR was performed as described in the above paragraph. The fold change was calculated and correlated with the RNA-seq data using the Pearson correlation coefficient in Microsoft Excel (version 2016 https://www.office.com/). The primers that were used in this study are listed in Supplementary Table S1. 

## 3. Results

### 3.1. Transcriptome-Wide Identification and Characterization of Stem Cell-Related Genes in Response to Nitric Oxide

**Figure 1:**
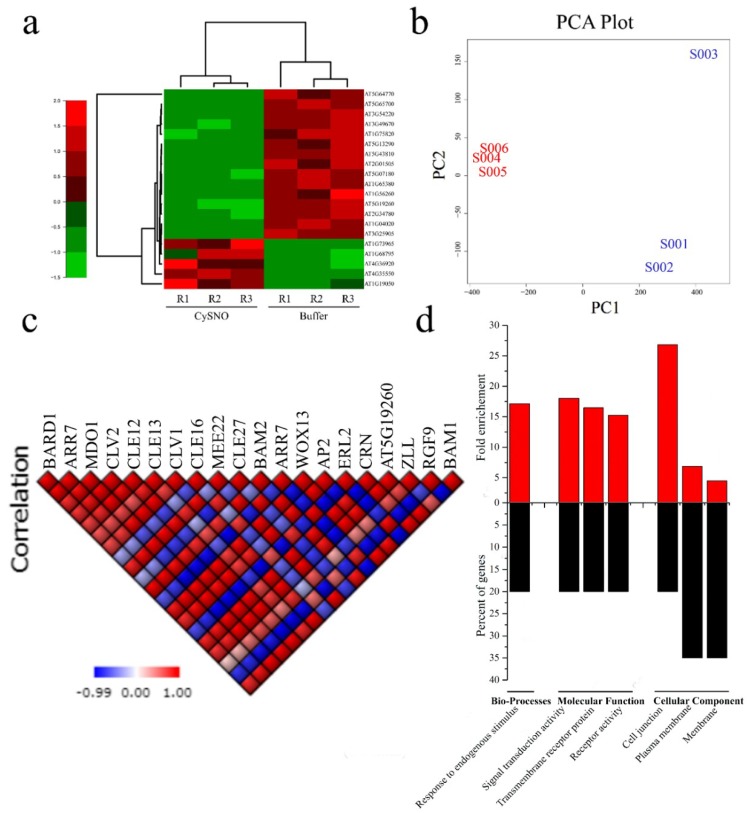
Identification and further analysis of nitric oxide (NO)-induced stem cell-related genes. (**A**) Heatmap showing expression patterns along with a dendrogram representing hierarchical clustering generated from the fragments per kilobase of transcript per million mapped reads (FPKM) values of the S-nitrosocysteine (CySNO)-induced stem cell-related genes. (**B**) A principal component analysis (PCA) plot showing dispersion in control and treated samples. S001–S003 in blue color represents replicates one to three control samples, whereas S004–S006 (red color) represents the CySNO-treated samples. Both panels (**A**,**B**) were generated using the R program. The color key in panel A represents the expression values from lowest (1.5) to highest (+2.0). (**C**) Pearson correlation among CySNO-induced stem cell-related genes and (**D**) gene ontology (GO) terms for biological processes, molecular functions, and cellular components.

To identify stem cell-related genes, we manually searched the list of all the differentially expressed genes (DEGs) in the CySNO-induced transcriptome [26]. We found approximately 20 different stem cell-related genes that showed differential response to one mM of CySNO (Figure 1A; Supplementary Table S2). A heatmap representing expression patterns and a dendrogram showing hierarchical clustering among the control and CySNO-treated samples (Figure 1A) were generated. A PCA plot showing the dispersion in data is also shown in Figure 1B. All of these genes showed at least a two-fold change (Supplementary Table S2). These genes included the *Breast Cancer-Associated Ring 1* (*BARD1*) that particularly binds with H3K4me3 of the target gene (such as *WUS*). *BARD1* is also required for organizing the shoot apical meristem and development of a quiescent center (QC) in the root apical meristem (RAM) [37,38]. Another important DEG was *Arabidopsis Response Regulator 7* (*ARR7*), which helps in the phosphorylation of the aspartic acid residue that induces the protein to activate the transcription of target genes. Similarly, we found *MERISTEM DISORGANIZATION 1* (*MDO1*), which was an uncharacterized protein that is required for the maintenance and organization of meristems by reducing DNA damage [39]. The *CLAVATA* genes were another important gene class in stem cell DEGs (Supplementary Table S2). The CLV1 protein, which is involved in signal regulation during meristem maintenance and regulates the balance between meristem cell proliferation and differentiation [40], and the CLV2 protein, which is a receptor-like protein involved in the perception of CLV3 and mediates organ and meristem development in *Arabidopsis* [41], showed response to the nitric oxide (NO) donor CySNO (Supplementary Table S2). We also found the CLE family members that are mostly involved in regulating root meristem maintenance. Among others were *SCARECROW*, which is a transcription factor that is required for the asymmetrical cell division of the cortex [42]; *WUSCHEL*-*related homebox 13* (*WOX13*); *APETALA 2* (*AP2*), which is required for early floral meristem identification and subsequently the transition of inflorescence meristem into floral meristem [43,44]; *CORYNE* (*CRN*), which is supposed to be involved in the CLV3 peptide in addition to the modulation of root, shoot, and flower meristem organization [45]; and *FANTASTIC FOUR 3* (*FAF3*), which that can repress *WUS* and influence shoot meristem size [46] (Supplementary Table S2). 

Interestingly, approximately 75% of the CySNO-induced stem cell-related genes were down-regulated. We further sought to determine a correlation among these genes, and selected the ones with high correlation coefficient factors (R ≥0.8). We found that the highest number of positive correlations were for *AT1G68795* (*CLE12*), which had positive correlations (R >0.8 unless stated otherwise) with 15 other genes, and negative correlations with three genes (Figure 1C). This was followed by *AT5G65700* (*BAM1*), which had 14 positive correlations with other genes (Figure 1C). Similarly, the lowest positive correlations were found for *AT5G43820* (*ZLL*), which encodes *Argonaute10* and showed only one positive correlation. Furthermore, the highest number of negative correlations was found for *AT3G54220*, which regulates the radial organization of the root, and for *AT5G64770* (*RGF9*), which encodes a root meristem growth factor; these each had negative correlations with 11 genes (Figure 1C). 

We further analyzed all 20 NO-responsive stem cell-related genes for the GO terms of biological processes, molecular function, and cellular components. In the GO terms for biological processes, we only found an association with one GO term, which was response to endogenous stimulus (Figure 1D). Similarly, in the GO terms for molecular functions, the highest category in terms of fold enrichment (*p*-value = 0.0000655) was signal transducer activity. Another category studied was the GO terms for cellular components, in which the majority of genes were associated with plasma membrane, while the highest fold enrichment was found for cell junction (Figure 1D). 

### 3.2. Promoter Anlaysis for Identification of Cis-Regulatory Elements

**Figure 2:**
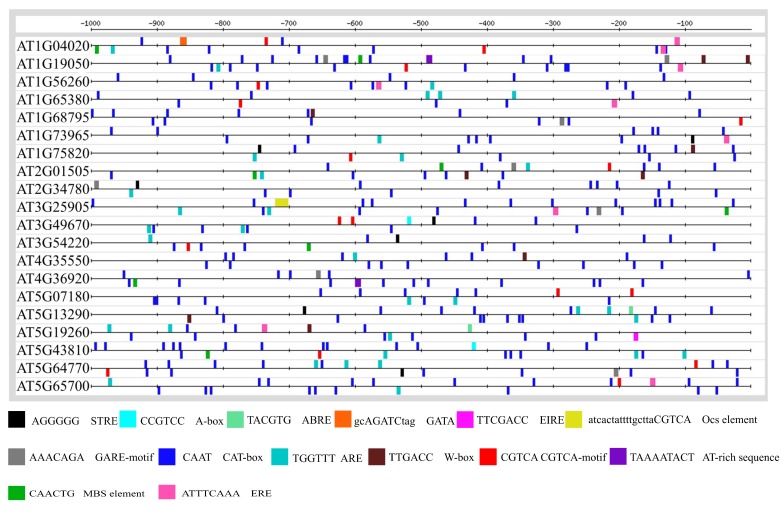
Promoter analysis of the CySNO-induced stem cell-related genes. Promoter sequences one Kb upstream of the transcription initiation site ATG were retrieved from The *Arabidopsis* Information Resource (TAIR; https://www.arabidopsis.org/) and analyzed for the identification of *cis*-regulatory elements. Only those *cis*-acting elements that were involved in biotic and/or abiotic stress conditions were mapped here.

*Cis*-regulatory elements are non-coding DNA sequences in the promoter of a gene that serve as potential binding sites of other regulatory proteins such as transcription factors (TFs) [47]. The presence of particular *cis*-regulatory elements in the promoter can predict the function of a gene, as these elements are reported to play a key role in the mechanistic control of the transcription of these genes [27,48]. Therefore, we were prompted to determine the *cis*-regulatory elements in the promoters of the CySNO-induced stem cell-related genes. For this, we analyzed the promoter region one Kb upstream of the transcription initiation site. About 14 *cis*-regulatory elements that were involved in growth and abiotic or biotic stress conditions were found. These elements included the CAT-box in 100% sequences; ARE in 90% sequences; CGTCA-motif in 65% sequences; STRE in 45% sequences; ERE in 40% sequences; GARE, W-box, and MBS in 35% sequences, A-box, ABRE, and AT-rich motif in 10% sequences, and GATA, EIRE, and OCS in 5% sequences (Figure 2). 

### 3.3. Phylogenetic Analsysis of Arabidopsis Stem Cells and their Orthologs in Other Species

**Figure 3:**
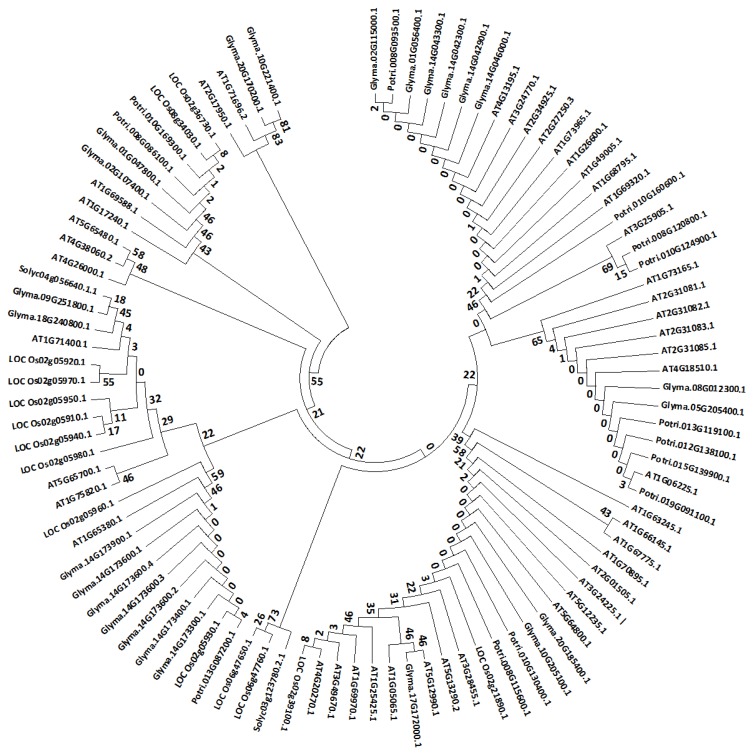
Molecular phylogenetic analysis of stem cell-related proteins. Analysis was performed using the stem cell-related protein sequences from *Arabidopsis*, rice, soybean, tomato, and poplar. The evolutionary history was inferred by using the maximum likelihood method based on the JTT (Jones Taylor-Thornton) matrix-based model [1]. The tree with the highest log likelihood (–34197.0000) is shown. The initial tree(s) for the heuristic search were obtained automatically by applying the neighbor-join (NJ) and BioNJ algorithms to a matrix of pairwise distances estimated using the JTT model, and then selecting the topology with a superior log likelihood value. The analysis involved 97 amino acid sequences. All of the positions with less than 95% site coverage were eliminated; that is, fewer than 5% alignment gaps, missing data, and ambiguous bases were allowed at any position, leaving only three positions in the final dataset. The evolutionary analyses were conducted in MEGA7 [2].

We were further interested in checking whether the selected stem cell-related genes were evolutionary conserved. Therefore, we retrieved the protein sequences of the stem cell-related genes from *Arabidopsis*, rice, soybean, tomato, and poplar, and analyzed them through MEGA 7.0 [31]. We found that most of the soybean and populus stem cell-related proteins were grouped together with the *Arabidopsis* CLE type of stem cells proteins; however, some of the soybean stem cell proteins were also grouped with the *Arabidopsis* receptor-like kinases such as BAM1 (*At5g65700*), CLV1 (*At1g75820*), and CLV2 (*At1g65380*) (Figure 3). Furthermore, the majority of rice stem cell-related proteins were grouped together with the *Arabidopsis* receptor-like kinase type of stem cell proteins (Figure 3). Tomato had the least number of stem cell-related proteins homologous to the *Arabidopsis* stem cell-related proteins, and they were grouped together with the *Arabidopsis* receptor-like kinase type of stem cell proteins (Figure 3). 

### 3.4. Interactome of CySNO-Induced Stem Cell-Related Genes

**Figure 4:**
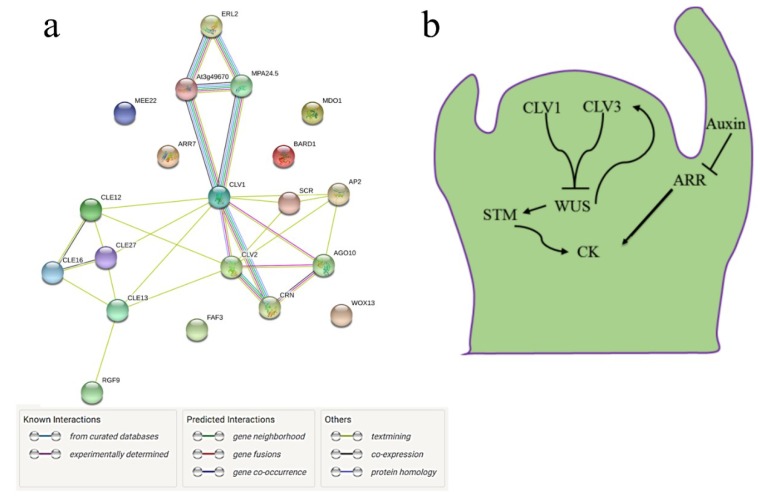
An interactome of CySNO-induced stem cell-related genes. (**A**) The interactome of stem cell-related genes that showed differential expression in response to CySNO was analyzed through STRING (https://string-db.org/cgi/network.pl). All kinds of predicted and known interactions are shown with different colors. (**B**) Predicted model of how stem cell-related genes regulate hormonal balance, specifically auxin and cytokinin regulation, in meristematic tissues.

Gene interaction is the key to understanding the functional relationships among genes or their corresponding gene products [49]. Therefore, we sought to determine the interaction of the CySNO-induced stem-cell related genes. Both experimentally confirmed and inferred interactions were studied using the Search Tool for the Retrieval of Interacting Genes/Proteins [STRING (https://string-db.org/)]. We found interesting interactions, specifically for the clavata protiens. The Clavata 1 (CLV1) proteins were found to interact with CLV2, BAM2, BAM1, and CRN. These interactions were predicted from the curated as well as experimentally proven databases (Figure 4A). Both BAM1 and BAM2 seem to be involved in a feedback loop regulating ERL2 that further regulates inflorescence and organ shape organization, including stomatal patterning (Figure 4A). The *CLAVATA* stem cell-related genes mostly work in coordination with hormonal pathways involved in growth and development, such as auxin and cytokinin. Previous reports suggested that the induction of *CLV1* and *CLV3* blocks *WUS*, which induces *CLV3* for meristem differentiation [9]. Furthermore, *WUS* positively regulates *SHOOTMERISTEMLESS*, which promotes cytokinin (CK) synthesis. High auxin levels can inhibit *ARABIDOPSIS RESPONSE REGULATOR* (*ARR*), resulting in the high levels of CK (Figure 4B). Thus, the complex coordination of genes is required to maintain meristem integrity and stability. 

### 3.5. CLV1 and CLV3 Differentially Regulate Nitrosative and Oxidative Stresses

**Figure 5:**
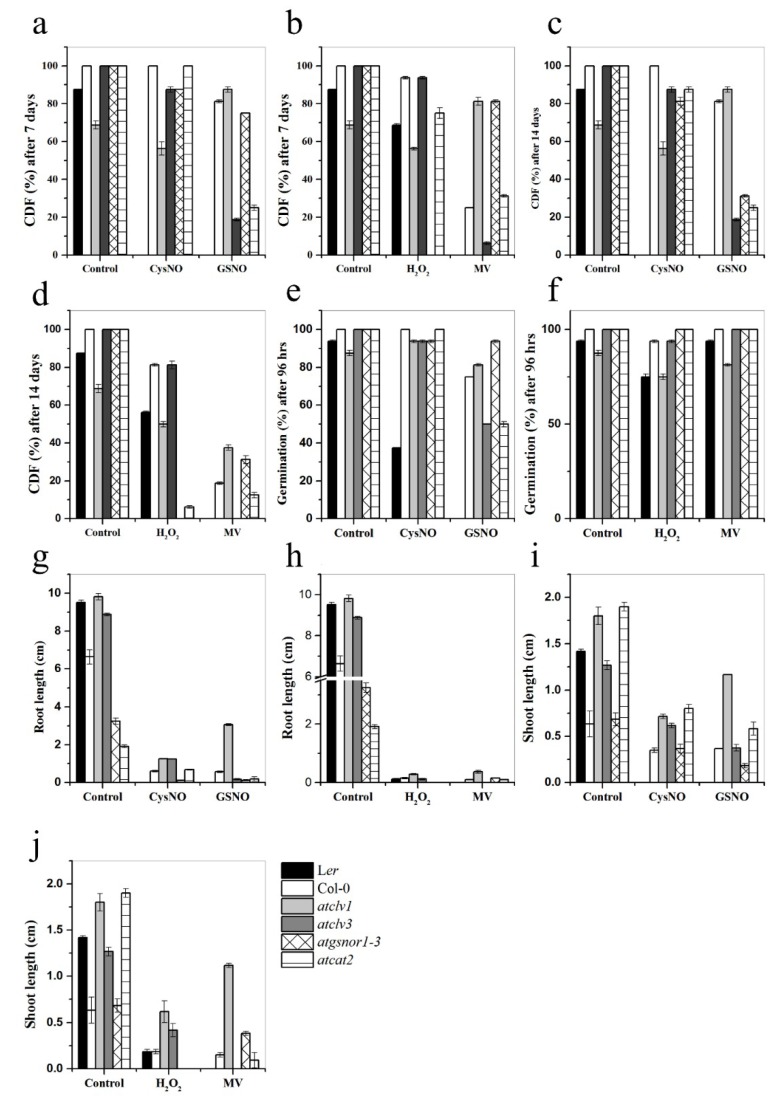
Response of the *clv1* and *clv3* plants exposed to oxidative and nitrosative stress conditions. The indicated genotypes were grown on the half Murashige and Skoog (MS) media supplemented with either CySNO or S-nitrosoglutathione (GSNO) for nitrosative stress conditions and with H_2_O_2_ and Methyl Viologen (MV) for oxidative stress conditions. (**A**) Cotyledon development frequency (CDF) of the indicated genotypes one week after sowing under nitrosative stress and (**B**) oxidative stress conditions. (**C**) CDF after two weeks of sowing under oxidative and (**D**) nitrosative stress conditions. (**E**) Percentage germination of the indicated genotypes under nitrosative and (**F**) oxidative stress conditions. (**G**) Root lengths of the indicated genotypes after two weeks under nitrosative and (**H**) oxidative stress conditions. (**I**) Shoot length under nitrosative and (**J**) oxidative stress conditions. All data points are the mean of at least three replicates, and experiments were repeated twice with almost similar results. The error bar represents the standard error (±SE). The Y-axis in panel ‘h’ was interrupted to clarify lower values.

The effect of redox-active molecules, such as NO and H_2_O_2_, on stem cells has not been investigated so far. Therefore, we were tempted to test the phenotypic performance of the *Arabidopsis clv1* and *clv3* mutant plants grown on the media supplemented with either CySNO and GSNO (for nitrosative stress) or H_2_O_2_ and MV (for oxidative stress), and obtained interesting results. First, the response of *clv1* and *clv3* to nitrosative as well as oxidative stress was significantly different from each other, as *clv3* was more resistant to the effects of CySNO and H_2_O_2_, but highly susceptible to GSNO and MV compared to *clv1*, which showed a completely opposite response, as indicated by the CDF measurements recorded after seven days of stress (Figure 5A,B). More interestingly, the resistant response of *clv3* and susceptible response of *clv1* was significantly higher than the known response of the *atgsnor1-3* plants. The same pattern of CDF was observed after 14 days of stress (Figure 5A,B). Although the germination percentage of *clv1* was similar to that of *clv3* on the CySNO media, a significantly higher number of *clv1* seeds germinated on the GSNO media, and less germinated on the media supplemented with H_2_O_2_ and MV compared to the *clv3* seeds after four days of sowing (Figure 5E,F). Although the root lengths of *clv1* and *clv3* plants were inherently shorter than those of the WT L*er* plants grown on control media, the root lengths of both the mutants were significantly reduced on both nitrosative and oxidative stress media (Figure 5G,H). On the other hand, the shoot lengths of the *clv1* and *clv3* plants were significantly higher than those of the WT L*er* plants grown on the stress media. Especially, the *clv1* plants produced significantly longer shoots on the media supplemented with CySNO, GSNO, H_2_O_2_, and MV (Figure 5I,J). Another interesting result was the completely different response of the WT L*er* and Col-0 ecotypes to all stress media, wherein the L*er* plants were found to be generally more resistant than the Col-0 plants (Figure 5). Furthermore, the different responses of the *clv1* and *clv3* plants to different nitrosative and oxidative stress donors suggest a significant redundancy in the function of *clv1* and *clv3*, as well as the different involvement of these two genes in the perception of different types of stresses.

### 3.6. CLV1 and CLV3 Positively Regulates Basal Defense at Early Time Points

**Figure 6:**
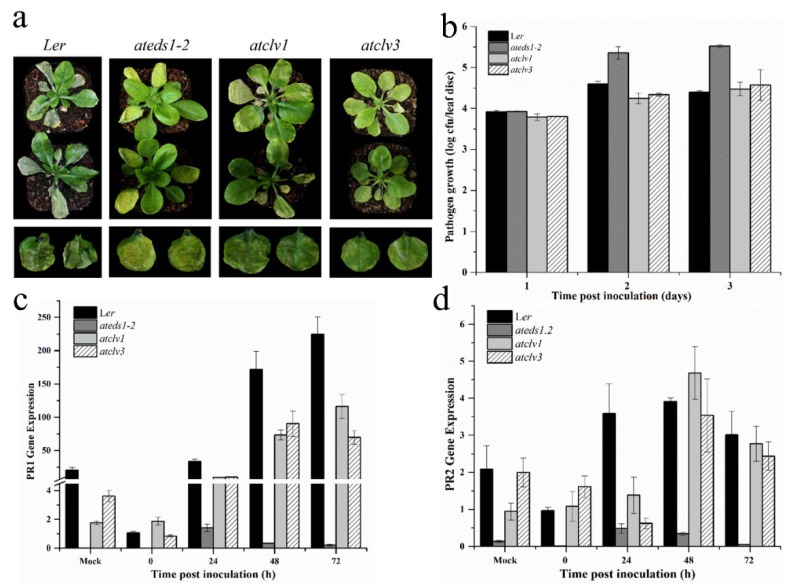
Both *CLAVATA* (CLV) mutants showed decreased *Pathogenesis-Related (PR*) gene expression after virulent pathogen inoculation. (**A**) Symptom development and general phenotypes of the indicated genotypes after *Pst* DC3000 (virulent) inoculation, (**B**) pathogen growth, (**C**) transcript accumulation of *PR1*, and (**D**) *PR2* expression in the indicated genotypes after *Pst* DC3000 inoculation. All data points are the means of the three replicates, and error bars represents ± standard error (SE) (*n* = 3). The background in panel ‘a’ was modified for more clarity, while the *Y*-axis in C was interrupted to visualize smaller values.

The characteristic phenotypic aberrations were observed for both these lines (*atclv1* and *atclv3*) under normal conditions as previously described by Clark et al. [50] and Koornneef et al. [51]. As these knock-out (KO) lines were in the Landsberg (L*er*) background, the WT and *eds1-2* L*er* plants were used as comparative controls. The results of the pathogenicity test showed that although both KO lines showed disease symptoms, they were significantly resistant to the *Pst* DC3000 infection compared to the WT and *eds1-2* L*er* plants, as indicated by the symptoms and colony counts after one and two days of inoculation. However, the bacterial CFU counts in both *clv* mutants after three days were slightly higher than those in the WT plants (Figure 6A,B). Interestingly, the qRT-PCR results showed that the expression of *PR1* and *PR2* did not correlate with the colony counts, as the expression of both these genes in the *clv1* and *clv3* plants was significantly lower that those in the WT plants after one, two, and three days of the *Pst*DC3000 inoculation (Figure 6C,D). This suggests that the resistance of the *clv* mutants to *Pst*DC3000 is either upstream or independent of *PR1* and *PR2* gene expression.

### 3.7. qRT-PCR Validation of RNA-Seq Data

**Figure 7:**
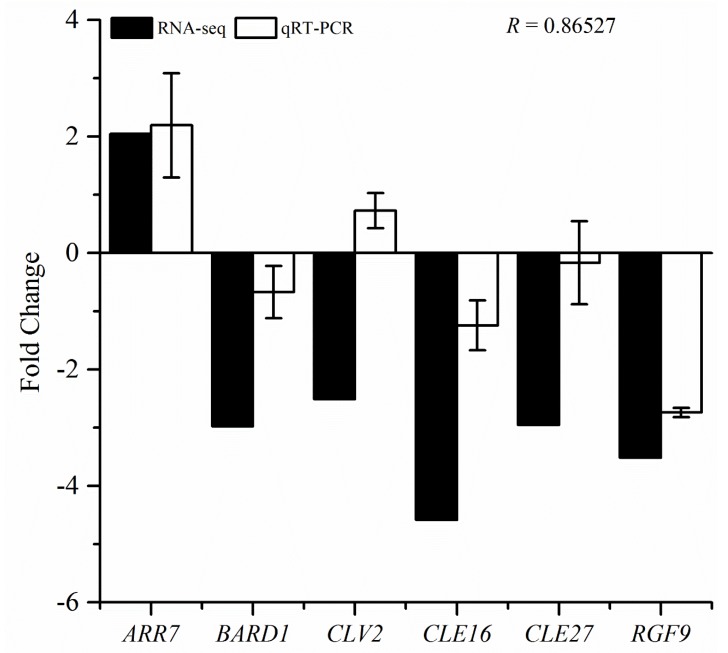
Validation of the RNA-seq analysis using qRT-PCR. Among the CySNO-induced stem cell-related genes, six were selected based on the fold change (Fold change >4) in their expression levels and analyzed through qRT-PCR. The transcript accumulation of these genes was compared with the RNA-seq data, and the Pearson correlation coefficient was calculated using Microsoft Excel. *R* represents the Pearson correlation coefficient. All of the data points are the means of the three replicates, and the error bars represent ± SE.

We further validated the transcriptional changes in the stem cell-related genes by selecting the representative genes and analyzing them through qRT-PCR. These genes were selected based on the fold change in their expression levels in response to treatment with one mM of CySNO. As the majority of the genes (75%) were down-regulated; therefore, we selected more down-regulated genes for validation analysis. We observed that out of the seven studied genes, six showed the same pattern as the RNA-seq results, except for the *CLV2* gene that was down-regulated in the RNA-seq results and up-regulated in the qRT-PCR analysis. We compared the datasets from both RNA-seq and qRT-PCR analyses by measuring the Pearson correlation coefficient and a found higher correlation (R = 0.865), indicating higher similarity among the two datasets (Figure 7). 

## 4. Discussion

Nitric oxide, a small gaseous molecule and newly emerged redox-active, is an important signaling component during both biotic and abiotic stress conditions in both animals and plants [16,52]. Therefore, it attracted the attention of plant scientists in the last couple of decades mainly because of its role in plants. Since then, it has been found to be a potent molecule affecting various physiological processes, such as seed germination, stomatal regulation, disease resistance, and plant responses to abiotic environmental conditions [15,16,53,54]. Its mechanism of action was unclear until it was found to transfer its bioactivity through *S*-nitrosylation, which is a post-translational modification in which NO covalently combines with the cysteine thiols of other proteins making *S*-nitrosothiols (SNOs) [55]. Thus, it can regulate physiological processes either by directly controlling gene transcription or by changing the redox status of cells by producing RNS. Studying global changes in gene expression in response to NO will make us understand the optimum genetic makeup during stress conditions through using various techniques such as microarray [56], amplified fragment length polymorphism [57], qRT-PCR [58], and RNA-seq [26,59]. 

Stem cell study is another trending topic in life sciences owing to their possible role in the medical field. Higher plants develop organs throughout their life; therefore, they must retain a pool of undifferentiated cells by regulating the proliferation of meristematic cells. In the *Arabidopsis* CLAVATA gene family, most genes are responsible for maintaining STM and OC [8,9]. Mostly, stem cells are associated with plant growth and development, and their role in plant disease resistance is poorly understood. Therefore, in the current study, we focused on stem cell or stem cell-related genes that showed differential expression in response to the NO donor *S*-nitroso-L-cysteine [26]. As NO is mostly produced in response to stress in plants [60], it would be interesting to determine the role of stem cell-related genes in plant growth and defense response. 

Among the 20 CySNO-induced stem cell-related genes, 75% were down-regulated, suggesting that most of the CySNO-responsive stem cell-related genes were either involved in the NO production pathways or in the negative feedback loop of genes that are positively regulated by NO. For example, *AtWOX13* (*AT4G35550*) that encodes a WUSCHEL-related homeobox gene family member is up-regulated, whereas both *CLV1* and *CLV2* were down-regulated in response to CySNO (Figure 1A; Supplementary Table S2). Reports suggested that *WUSCHEL* (*WUS*) is required for stem cell identity, whereas the *CLV* genes regulate the pathway that limits the size of the shoot meristem. Furthermore, *CLV1* and *CLV3* suppress *WUS* expression to maintain the size of the stem cell population in the shoot meristem. The knock-out (KO) lines *clv1* and *clv3* showed irregular growth patterns; thus, *CLV* and *WUS* work in a negative feedback loop to maintain the OC [61]. Similarly, another gene *BARD1* (*AT1G04020*) was down-regulated in response to CySNO (Figure 1A; Supplementary Table S2). Interestingly, *BARD1* is also implicated in the regulation of *WUS* (up-regulated to CySNO) expression. Han, Li, and Zhu [37] reported that *BARD1* regulates SAM organization and OC maintenance by limiting WUS expression to the OC. They confirmed through functional genomics that the *bard1-3* mutants resulted in severe SAM defects, while the double mutants *wus-1* and *bard1-3* resulted in the prematurely terminated SAM structures, suggesting that *BARD1* functions through the regulation of *WUS* [37]. 

A correlation among the CySNO-induced stem cell-related genes suggested that the highest positive correlation (>0.8) was found for *CLE12*, which had positive correlations with 15 genes, followed by *BAM1* (Figure 1C). The CLE gene family comprises 32 members of Arabidopsis, and members of this family are involved in vital growth processes, such as root growth, rosette growth, root dwarfing, SAM arrest, asymmetric leaf development, etc. [10]. Most of the CLE gene family members are involved in the regulation of root growth with differential effects [10]. The GO terms for molecular function revealed that the major GO terms to which the stem cell-related genes were associated were related to signaling, receptor activity, and transmembrane receptor protein (Figure 1D). This might be because a strong signaling capacity is required for the stem cell-related genes to maintain SAM. This can be confirmed by the role of *CLV* in maintaining SAM through the suppression of *WUS,* as supported by the reports suggesting that the CLV3 peptide is perceived by at least four different receptor-like proteins to repress the WUS activity [9,40]. The analysis of the promoter regions for *cis*-regulatory elements has suggested the presence of *cis* elements involved in abiotic and biotic stress tolerance. These include ABRE (TACGTG), which is involved in osmotic stress response and drought stress regulation [62]; TATA-box, a basic promoter element for enhanced transcription [63]; EIRE (TTCGACC), which is involved in maximal elicitor-mediated activation [64]; and MBS, the promoter element that harbors the transcription factor (TF) binding site for the MYB TF [65]. In addition, the W-box (TTGACT/C), which is the binding site for the WRKY TF, is considered to be involved in the regulation of multiple regulatory pathways involved in both abiotic and biotic stresses [27]. Previous studies also suggested the presence of the TF binding sites in NO-responsive genes. Through a bioinformatics approach, Palmieri et al. [66] have analyzed approximately 28447 *Arabidopsis* genes, and found that several TF binding sites occurred at least 15% more often in the promoters of NO-induced genes. 

A phylogenetic analysis revealed that the majority of the soybean and poplar stem cell-related proteins were grouped together with the *Arabidopsis* CLE type of stem cell proteins (Figure 3). Similarly, a majority of the soybean stem cell-related genes were grouped together with the *Arabidopsis* receptor-like kinases type of stem cell proteins, such as BAM1 (*At5g65700*), CLV1 (*At1g75820*), and RLP12 (*At1g71400*) (Figure 3), indicating that the stem cell-related proteins of *Arabidopsis* and soybean share more common features than those of the other species. Furthermore, it was suggested that the types of stem cells might vary depending upon the types of species and their life forms. 

An interactome study of the CySNO-induced stem cell-related genes suggested both experimentally proven and computationally inferred protein interactions. Among them, CLV1, CLV2, and CRN are important stem cell proteins that are involved in the maintenance of stem cells. Both CLV2 and CRN are involved in the perception of CLV3 (Figure 4A). An extensive in vivo analysis has proven that CLV1 and CLV3 interact to repress WUS expression in order to maintain stem cell organization [8,9,40]. *CLAVATA* also works in coordination with phytohormones that are involved in growth and development. Reports suggested that *WUS* positively regulates STM, which promotes cytokinin synthesis. Similarly, high auxin levels can block the expression of *ARR*, resulting in high cytokinin levels (Figure 4B). 

Further investigation into the physiological roles of *CLV1* and *CLV3* indicated that under control conditions, both *clv1* and *clv3* responded differently. However, under the CySNO-induced nitrosative stress condition, both *clv1* and *clv3* plants showed increased root and shoot length compared to the wild-type plants (Figure 5G,I). This indicates that both *CLV1* and *CLV3* negatively regulate shoot and root growth under CySNO stress conditions. *CLV1* (*At1g75820*) was down-regulated in response to CySNO (Supplementary Table S2), which implies that CySNO negatively regulates the *CLV1* expression; therefore, an opposite situation would be expected from the *clv1* KO mutant. Furthermore, *CLV1* in combination with *CLV3* inhibits the *WUS* expression; therefore, *clv1* would be unable to repress *WUS*, which induces STM to promote the production of cytokinin [40,67], which is an important growth-inducing hormone. 

Another interesting aspect of both *clv1* and *clv2* mutants was to study their role in the basal defense system. Interestingly, *clv1* and *clv3* showed resistant phenotypes, and showed less pathogen growth, specifically at two days post-inoculation (Figure 6B); however, the *PR1* and *PR2* gene expression study did not reflect the resistant phenotype. Our results suggested decreased *PR1* and *PR2* transcript accumulation in response to virulent pathogens, suggesting that the resistance toward *Pst*DC3000 might be upstream or independent of the SA pathway. However, further investigation is required in order to explore the exact mechanism. We also validated the RNA-seq-mediated transcriptional changes in the CySNO-induced stem cell-related genes and correlated these with qRT-PCR. The Pearson correlation coefficient of 0.86527 indicates the high reliability of the RNA-seq data. 

This study will add to our understanding of the role of NO in stem cell-mediated growth and defense response toward virulent pathogens.

## 5. Conclusions

This study concludes that nitric oxide-induced stem cell related genes are conserved across the plant kingdom, as they have orthologs in other species such as rice, soybean, tomato, and poplar plants. The promoter study suggested their putative role in plant defense; however, in vivo analysis using the inoculation of mutant lines *clv1* and *clv3* with virulent *Pst*DC3000 showed a resistant phenotype, but reduced *PR* gene expression. Furthermore, stem cell-related genes differentially regulated nitrosative and oxidative stress. Together all of the NO-induced stem cell-related genes may directly regulate plant growth, but indirectly regulate the plant defense system. 

## Supplementary Materials

The following are available online at https://www.mdpi.com/2073-4425/10/3/190/s1, Supplementary Table S1: List of primers used in this study, Supplementary Table S2: List of stem cell-related DEGs in response to 1mM CySNO.
