# Supplementary material for: Comprehensive Analyses of Nitric Oxide-Induced Plant Stem Cell-Related Genes in Arabidopsis thaliana
Source: Genes (Basel). 2019 Mar 1;10(3):190. doi: 10.3390/genes10030190 (PMC6471024; doi:10.3390/genes10030190)
Supplement: Supplementary file 1 [file genes-10-00190-s001.zip › Supplementary files/Supplementary Table S1.docx]

| Accession | Name | Primer F (5´-3´) | Primer R (5´-3´) |
| --- | --- | --- | --- |
| AT1G19050 | *ARR7* | GGTTGGTGAGGTCATGAGGA | CTGCAAAGCCCTAGTTCCAC |
| AT1G73965 | *CLE13* | GAAGAGCTTTGGCGGTGAAA | ACGCCATATCTCGGGTCAAT |
| AT1G04020 | *BARD1* | ATCCAGGAAAGCAGTCCGAA | CATTGCATCAGAAGCGGTGA |
| AT1G65380 | *CLV2* | TCGTCTTCTCTCCCTCGTTG | ATGAGCCGTACCATGTCGAT |
| AT1G75820 | *CLV1* | TCAATCCCTACCGGAATCGG | TTGGACAAGAGACACGGTGA |
| AT2G01505 | *CLE16* | AGACGAGCATACACCACCAG | TGACCAGCCTTTCTCGGTAG |
| AT3G25905 | *CLE27* | TTTCCAGAAACTCCGGCTTC | AAGGATCCGGACAACTTGGT |
| AT2G14610 | *PR1* | GTGCAATGGAGTTTGTGGTC | TCACATAATTCCCACGAGGA |
| AT3G57260 | *PR2* | CAGATTCCGGTACATCAACG | AGTGGTGGTGTCAGTGGCTA |
| AT3G18780 | *Actin 2* | GCTGGACGTGACCTTACTGA | CCATCTCCTGCTCGTAGTCA |

Table. S1. List of primers used in this study
